# RNA from LPS-stirnulated macrophages induces the release of tumour necrosis factor-α and interleukin-1 by resident macrophages

**DOI:** 10.1155/S0962935193000626

**Published:** 1993

**Authors:** R. A. Ribeiro, F. L. De Lucca, C. A. Flores, F. Q. Cunha, S. H. Ferreira

**Affiliations:** 1 Department of Pharmacology Faculty of Medicine of Ribeirão Preto University of São Paulo Ribeirão Preto S.P. Brazil; 2 Department of Biochemistry Faculty of Medicine of Ribeirão Preto University of São Paulo Ribeirão Preto S.P. Brazil

## Abstract

The effect of exogenous RNA on many cellular functions has been studied in a variety of eukaryotic cells but there are few reports on macrophages. In the present study, it is demonstrated that cytoplasmatic RNA extracted from rat macrophages stimulated with Escherichia coli lipopolysaccharide (LPS), referred to as L-RNA, induced the release of TNF-α and IL-1 from monolayers of peritoneal resident macrophages. The activity of L-RNA was not altered by polymyxin B but was abolished by ribonuclease (RNase) pretreatment, indicating the absence of LPS contamination and that the integrity of the polynucleotide chain is essential for this activity. Both the poly A(−) and poly A(+) fractions obtained from L-RNA applied to oligo(dT)–cellulose chromatography induced TNF-α and IL-1 release. The L-RNA-induced cytokine release was inhibited by dexamethasone and seemed to be dependent on protein synthesis since this effect was abolished by cycloheximide or actinomycin-D. The LPS-stimulated macrophages, when pre-incubated with [5-^3^H]-uridine, secreted a trichloroacetic acid (TCA) precipitable material which was sensitive to RNase and KOH hydrolysis, suggesting that the material is RNA. This substance was also released from macrophage monolayers stimulated with IL-1β but not with TNF-α, IL-6 or IL-8. The substance secreted (^3^H-RNA) sediments in the 4–5S region of a 5–20% sucrose gradient. These results show that L-RNA induces cytokine secretion by macrophage monolayers and support the idea that, during inflammation, stimulated macrophages could release RNA which may further induce the release of cytokines by the resident cell population.


Mediators of Inflammation 2, 435–442 (1993)

### RNA from LPS-stimulated macrophages induces the release of tumour necrosis factor-α and interleukin-1 by resident macrophages

R. A. Ribeiro,[1] F. L. De Lucca,[2,CA] C. A. Flores,[1] F. Q. Cunha[1] and S. H. Ferreira[1]

[1]Departments of Pharmacology and [2]Biochemistry, Faculty of Medicine of Ribeirão Preto, University of São Paulo, Ribeirão Preto, S.P., Brazil

[CA] Corresponding Author

**THE effect of exogenous RNA on many cellular functions has been studied in a variety of eukaryotic cells but there are few reports on macrophages. In the present study, it is demonstrated that cytoplasmatic RNA extracted from rat macrophages stimulated with *Escherichia coli* lipopolysaccharide (LPS), referred to as L-RNA, induced the release of TNF-α and IL-1 from monolayers of peritoneal resident macrophages. The activity of L-RNA was not altered by polymyxin B but was abolished by ribonuclease (RNase) pretreatment, indicating the absence of LPS contamination and that the integrity of the polynucleotide chain is essential for this activity. Both the poly A(−) and poly A(+) fractions obtained from L-RNA applied to oligo(dT)–cellulose chromatography induced TNF-α and IL-1 release. The L-RNA-induced cytokine release was inhibited by dexamethasone and seemed to be dependent on protein synthesis since this effect was abolished by cycloheximide or actinomycin-D. The LPS-stimulated macrophages, when pre-incubated with [5-$^3$H]-uridine, secreted a trichloroacetic acid (TCA) precipitable material which was sensitive to RNase and KOH hydrolysis, suggesting that the material is RNA. This substance was also released from macrophage monolayers stimulated with IL-1β but not with TNF-α, IL-6 or IL-8. The substance secreted ($^3$H-RNA) sediments in the 4–5S region of a 5–20% sucrose gradient. These results show that L-RNA induces cytokine secretion by macrophage monolayers and support the idea that, during inflammation, stimulated macrophages could release RNA which may further induce the release of cytokines by the resident cell population.**

**Key words:** Exogenous RNA, Interleukin-1, LPS, Macrophage, RNA secretion, Tumour necrosis factor-α

### Introduction

There is considerable evidence indicating that exogenously supplied RNA molecules can be incorporated into eukaryotic cells and can exert a variety of biological effects both *in vitro* and *in vivo*.[1–5] Cell differentiation, genetic transformation and immunological effects induced by exogenous RNA have been investigated over the past few years. For example, RNA isolated from the endoderm of normal embryos of the amphibian *Ambystoma mexicanum* causes the differentiation of mutant heart tissue into functional, contractile cardiac muscle.[6] The genetic nutritional deficiencies of a strain of *Aspergillus nidulans* have been corrected by genetic transformation using RNA extracted from the wild strain of this fungus.[7] Recently, the authors demonstrated the transfer, to human lymphocytes, of cellular immunoreactivity to a highly conserved and immunodominant, synthetic epitope of the glycoprotein 41 of HIV-1, by using RNA isolated from animals immunized with this synthetic peptide.[8]

The effect of exogenous RNA has also been detected *in vivo*. The direct injection of purified mRNA for the bacterial enzyme chloramphenicol acetyl transferase (CAT) into mouse skeletal muscle results in significant CAT activity in these muscle cells.[9] Similarly, the injection of synthetic mRNA for the Wnt oncogene family induces cellular differentiation in *Xenopus* embryos, associated with the formation of specific embryonic structures.[10] The RNA obtained from the lymphoid organs of animals immunized with carcinoma antigens from human colon induces memory T-lymphocytes against these tumour antigens *in vivo*.[11]

It is now becoming clear that the cytokines constitute a link between cellular injury or recognition of non-self and the development of the inflammatory and/or immune response. Among the cytokines, interleukin 1 (IL-1) and tumour necrosis factor-α (TNF-α) play an important role in triggering and in the maintenance of these events.[12,13] Although several cells release cytokines, the resident macrophages can be considered the

© 1993 Rapid Communications of Oxford Ltd most active cytokine secreting cells.[14,15] When activated by exogenous and/or endogenous stimuli, these macrophages release various cytokines, including TNF-α, IL-1, IL-6 and IL-8, which induce the local and systemic events of the defence response.[16,17]

Recently, it has been suggested that RNA may function as an intercellular mediator, acting over short distances, leading to the possibility of the transfer of biological information from one cell to another via RNA molecules.[18]

In the present study, we investigated whether RNA extracted from LPS-activated macrophages could stimulate the release of TNF-α and IL-1 from naive macrophages in culture. We also investigated whether macrophages stimulated by endotoxin released RNA into the culture medium. The results of the present investigation are consistent with the idea that RNA may be involved as an intercellular modulator of cytokine release from macrophages.

### Materials and Methods

*Preparation of macrophage monolayers:* Rat peritoneal cavities were stimulated by the injection of a 3% thioglycollate solution (10 ml/animal). Four days later, the peritoneal macrophages were harvested in RPMI medium and allowed to adhere to plastic tissue culture dishes for 1 h at 37°C, in an atmosphere of air containing 5% $CO_2$. The monolayers were then washed three times with phosphate buffered saline (PBS), pH 7.4, and incubated for 1 h at 37°C in RPMI medium containing 10 μg/ml of *Escherichia coli* lipopolysaccharide (LPS). The supernatants were discarded and the monolayers washed a further three times with PBS. The cells were harvested with a disposable rubber policeman and were used for RNA extraction after centrifugation (1000 × ***g*** for 10 min).

*RNA extraction:* Cytoplasmic RNA was extracted from LPS-stimulated macrophages using the cold phenol method described by White and De Lucca.[19] The macrophage pellet was homogenized in Tris-HCl buffer, pH 9.0, containing 0.01 M KCl and 0.25 M sucrose (2.5 ml/g macrophage pellet, wet weight) and centrifuged at 12 000 × ***g*** for 15 min at 4°C. The supernatant was diluted two-fold with the homogenization buffer and used as the source of cytoplasmic RNA.

The supernatant was deproteinized by the addition of an equal volume of 88% (v/v) phenol containing 0.05% bentonite. The mixture was shaken for 10 min and then centrifuged at 2 000 × ***g*** for 10 min at 4°C. The phenol phase was discarded and the phenol treatment repeated twice. An equal volume of 88% phenol–chloroform (1 : 1, v/v) solution was added to the aqueous phase of the phenol treatment. The mixture was shaken for 5 min at room temperature and centrifuged at 2 000 × ***g*** for 10 min at 4°C. The RNA was precipitated by the addition of crystalline NaCl to the final aqueous phase, to a final concentration of 0.1 M, plus 2.5 volumes of cold 96% ethanol. The solution was left for 18 h at −20°C and the precipitated RNA was collected by centrifugation and stored under ethanol at −20°C until use.

The concentration of RNA was calculated by measuring absorbance at 260 nm (1 absorbance unit = 40 μg of RNA/ml). Only those preparations of RNA with a ratio $A_{260}/A_{280}$ of about 1.9–2.0 were used. The RNA preparations obtained from LPS-stimulated macrophages are referred to as L-RNA. Samples of cytoplasmic RNA obtained from non-stimulated macrophage monolayers by the same extraction procedure are referred to as normal RNA (N-RNA).

The integrity of the RNA was routinely evaluated using SDS–polyacrylamide gel electrophoresis according to Bertolini and De Lucca.[20] The gels were fixed in acetic acid and scanned for absorbance at 260 nm in a spectrophotometer equipped with a linear transport.

*Oligo(dT)–cellulose chromatography:* The poly(A)-containing RNA (poly A(+)-RNA) and the poly(A)-lacking RNA (poly A(−)-RNA) fractions were separated from the bulk of the cytoplasmic RNA (total RNA) by affinity chromatography on an oligo(dT)–cellulose column as described by Aviv and Leder.[21]

*Enzymatic treatment of L-RNA:* The L-RNA preparations were subjected to enzymatic treatment using a mixture containing ribonuclease $T_1$ (EC 3.1.27.3; guanyloRNase) and bovine pancreatic ribonuclease (EC 3.1.27.5; RNase type I-A). The enzymes were diluted in 0.85% NaCl in the ratio of 1 μg of each RNase to 10 μg L-RNA and the mixture incubated at 37°C for 30 min.

*Polymyxin B treatment of L-RNA:* Samples of L-RNA (500 μg/ml) were preincubated at 37°C for 30 min with polymyxin B (100 μg/ml). The RNA was recovered by ethanol precipitation followed by centrifugation (2 000 × ***g***, at 4°C for 25 min). Samples of LPS (10 μg/ml) were also preincubated with polymyxin B.

*Treatment of macrophage monolayers with L-RNA or N-RNA:* Macrophage monolayers (2 × $10^7$ cells/well) obtained as described previously were cultured in 2 ml of RPMI medium supplemented with 10% foetal calf serum, 2 mM L-glutamine, 100 units/ml penicillin, and 100 μg/ml streptomycin, for 2 h at 37°C in an atmosphere of air containing 5% $CO_2$. The non-adherent cells were then removed by washing with RPMI medium. Two of the following stimuli were added to each well: LPS (5 μg/ml); L-RNA (25 μg/ml); L-RNA (25 μg/ml) submitted to enzymatic or polymyxin B pretreatment; N-RNA (25 μg/ml); poly A(+)-L-RNA (0.5 μg/ml), and poly A(−)-L-RNA (25 μg/ml). After 90 min incubation at 37°C, the supernatants were discarded and the cells washed three times with RPMI, followed by a final 6 h incubation period in RPMI medium (1.5 ml/well). For TNF or IL-1 assay, the cell-free incubation fluids were subsequently filtered through 0.22 μm Millipore membranes and stored at −70°C until tested. Control wells were incubated with RPMI medium alone.

To investigate the effects of actinomycin-D (10 μg/ml), cycloheximide (10 μg/ml) and dexamethasone (3 μg/ml) on the release of TNF and IL-1 by L-RNA-stimulated macrophage monolayers, the drugs were added to the incubation medium 30 min before the stimuli and throughout the stimulation period.

*TNF activity assay:* The TNF content of the RNA macrophage supernatants was measured using a highly TNF-sensitive cell line, WEHI 164 clone 13, as described elsewhere.[22] Briefly, WEHI cells were seeded in 96-well microplates at a concentration of $4 \times 10^4$ cells/well in 50 μl of supplemented RPMI. Fifty μl of serially diluted crude supernatant were added in quadruplicate to the cells. The plates were incubated for 20 h at 37°C in a 5% $CO_2$–air incubator. Ten μl of 3-[4,5-dimethylthiazol-2-yl]-2,5-diphenyltetrazolium bromide (MTT) solution (5 mg/ml PBS) were added to each well and the plates incubated for an additional 4 h. One hundred μl of isopropanol containing 0.04 N HCl were then added to each well. Fifteen min later, the degree of cell lysis was evaluated spectrophotometrically (570 nm) using an enzyme-linked immunoassay analyser.

Owing to the unavailability of rat TNF, standard curves were obtained with mrTNF-α; the TNF content of the assayed material was calculated by comparison with standard amounts. The results are given as the means of data obtained from at least three different experiments. In some experiments, a rabbit anti-mrTNF antibody was used to demonstrate the specificity of the assay. The results are expressed in international units (IU) of TNF/ml.

*IL-1 activity assay:* The IL-1 concentrations in supernatants of RNA-stimulated macrophage monolayers were measured using the thymocyte co-mitogenic assay.[23] Briefly, thymocytes obtained from the thymus of C3H/HeJ mice were plated in 96-well microplates at concentrations of $5 \times 10^5$ cells/well, in 100 μl of supplemented RPMI (see above). One hundred μl of serially diluted crude supernatant, with or without concanavalin A (Con A; 0.1–1 μg/well), were added to the incubation medium, and the plates incubated for 72 h at 37°C in a 5% $CO_2$–air incubator. [$^3$H]-Thymidine (0.5 μCi/well) was then added and the plates incubated for an additional 8 h. IL-1 concentrations were calculated by interpolating the values from standard curves constructed with rhuIL-1β (0.2–20 IU/well). The results are expressed as IU of IL-1/ml.

*Secretion of RNA by macrophages stimulated with LPS or cytokines:* The detection of RNA in the supernatants of macrophage monolayers was performed using [5-$^3$H]-uridine as a specific precursor of RNA synthesis, according to Kolodny *et al.*[24] Peritoneal macrophages, obtained from thioglycollate treated rats, were cultured in 12-well tissue culture plates at a concentration of $2 \times 10^7$ cells/well, in 2 ml of supplemented RPMI medium. The cells were allowed to adhere for 1 h at 37°C in a 5% $CO_2$–air incubator. The nonadherent cells were removed by washing with RPMI medium, and the macrophage monolayers were incubated with 2 ml of medium containing [5-$^3$H]-uridine (5 μCi/ml) for 2 h. The supernatants were then discarded and the cells washed three times with RPMI medium. To each well 2 ml of the following stimuli were added: LPS (0.1–10 μg/ml), rhuIL-1β ($10^{-10}$–$10^{-8}$ M), rhuTNF-α ($10^{-10}$–$10^{-8}$ M), rhuIL-6 ($10^{-10}$–$10^{-8}$ M) and rhuIL-8 ($10^{-10}$–$10^{-8}$ M). Control wells were incubated with RPMI medium alone. The cell cultures were then incubated for a final 6 h period. After incubation, the supernatants were removed and centrifuged at 10 000 × ***g*** for 30 min. The RNA was precipitated with 25% TCA at 4°C for at least 1 h. The precipitated radioactive material was filtered on a Millipore system using 0.45 μm membranes, followed by washing with 5% TCA. Finally, the radioactivity present in the membranes was detected in a liquid scintillation counter. Cell viability was determined by measuring lactate dehydrogenase (LDH) levels in the culture supernatants.[25]

*Treatment of $^3$H-RNA with RNase and KOH:* For each ml of $^3$H-RNA sample, 200 μl of 0.3 N KOH or 17 μl of a mixture of ribonucleases (2 mg of RNase type I-A + RNase $T_1$ 340 058 IU/mg of protein) were added. The tubes containing KOH were boiled for 45 min, and those treated with the enzymes were incubated at 37°C for 30 min. The samples were then processed for $^3$H-RNA detection as described previously.

*Sedimentation profile of $^3$H-RNA:* The $^3$H-RNA present in the supernatants from LPS-stimulated macrophage monolayers was treated with KOH or left untreated, followed by cold ethanol precipitation for at least 16 h. The $^3$H-RNA from unstimulated macrophage monolayers was precipitated with ethanol alone. The $^3$H-RNAs were collected by centrifugation (10 000 × *g* at 4°C for 25 min) and resuspended in 0.05 M acetate buffer, pH 5.0. Samples (100 μl) were layered on linear 5–20% sucrose gradients prepared in 0.05 M acetate buffer, pH 5.0, containing 0.005 M EDTA. The gradients were centrifuged at 53 000 × *g* for 5 h at 4°C and fractionated using a density gradient fractionater. The radioactivity of fractions collected was quantified using a liquid scintillation spectrometer.

*Materials:* Thioglycollate medium and lipopolysaccharide from *E. coli* (0111 : B4) were purchased from Difco Laboratories (Detroit, USA); RPMI 1640 medium from Flow Laboratories, USA; foetal calf serum (FCS), L-glutamine, streptomycin, penicillin, polymyxin B sulfate, bovine ribonuclease $T_1$, pancreatic ribonuclease, oligo(dT)–cellulose, MTT, concanavalin-A, FMLP, ribonuclease $T_1$ (EC 3.1.27.3; guanyloRNase) and bovine pancreatic ribonuclease (EC 3.1.27.5; RNase type I-A) from Sigma Co., USA; bis-acrylamide, acrylamide (Merck); recombinant human IL-1β, rhIL-6, rhIL-8, rhTNF-α were from the National Institute for Biological Standards (Herts, UK). WEHI 164 clone 13 cells and rmuTNF-α were a gift from Dr M.A. Palladino Jr. (Genentech Inc., USA). The anti-rmuTNFα antibody was a gift from Dr Wirla Tamashiro (State University of Campinas, Brazil). $^3$H-Thymidine and [5-$^3$H]-uridine were from Amersham, UK.

### Results

The preincubation of macrophage monolayers with cytoplasmic RNA (25 μg/ml) extracted from LPS (10 μg/ml) stimulated macrophages (L-RNA) caused a significant release of TNF and IL-1 in the culture medium as compared with the RNA from non-stimulated macrophages (N-RNA), or with RPMI medium alone (Fig. 1). Such cytokine release was abolished by treatment of the L-RNA samples with RNase but not with polymyxin B. Polymyxin B at the same dose abolished the ability of LPS to stimulate the release of TNF and IL-1. The integrity of our RNA samples was confirmed by SDS–polyacrylamide gel electrophoresis (data not shown).

The release of TNF (Fig. 2) and IL-1 (Fig. 3) by macrophages was induced by both poly A(+) and poly A(−) RNA fractions obtained by chromatography of the L-RNA (total L-RNA) on oligo (dT)–cellulose columns. Although the release of the cytokines was of similar magnitude, the dose of poly A(+) used was 50-fold less than that of poly A(−) or total L-RNA. Figure 2a shows that the TNF activity detected in supernatants of L-RNA

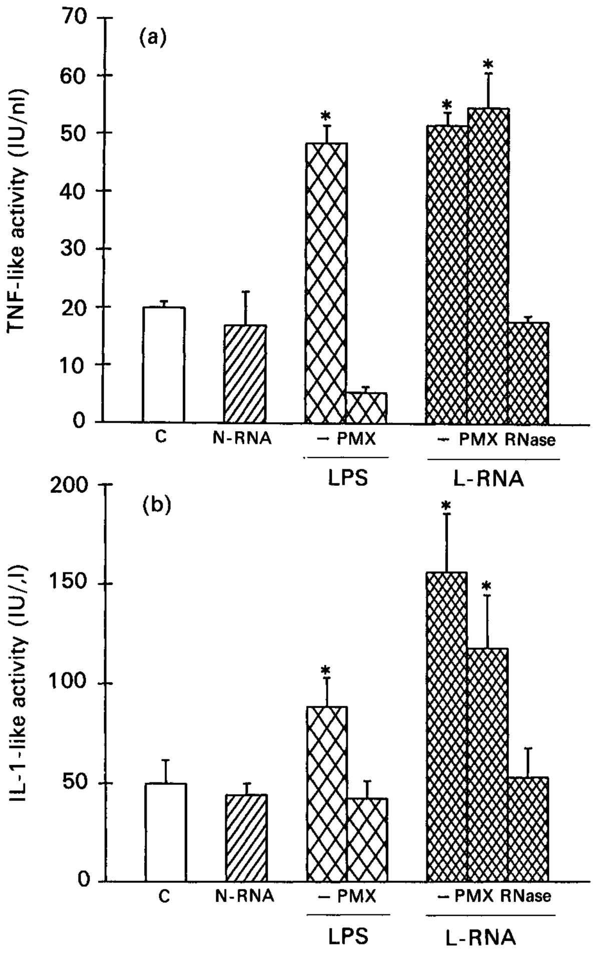

FIG. 1. The effect of L-RNA and N-RNA on the release of TNF-α and IL-1 by resident macrophages. Macrophage monolayers were incubated with N-RNA (25 μl/ml), L-RNA (25 μg/ml), LPS (10 μg/ml) or RPMI medium alone (C) for 6 h. The L-RNA was also incubated with RNase (2 μg of enzyme/10 μg of L-RNA) or PMX (100 μg/ml); LPS was used as a positive control. The activity of TNF-α (panel A) and IL-1 (panel B) was quantified as described in Materials and Methods. Results are given as the mean ± S.E.M. of quadruplicates from a representative assay taken from three similar experiments. *$p < 0.01$ compared with control value (ANOVA; Duncan's test).

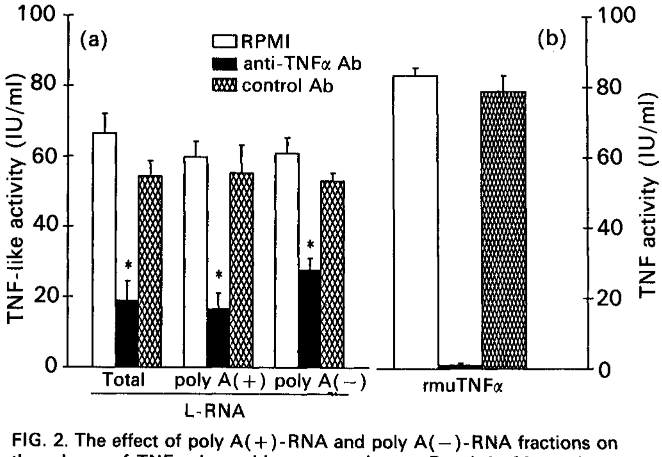

FIG. 2. The effect of poly A(+)-RNA and poly A(−)-RNA fractions on the release of TNF-α by resident macrophages. Panel A: Macrophage monolayers were incubated with total L-RNA (25 μg/ml), or with the poly A(+)- (0.5 μg/ml) or poly A(−)-RNA fractions (25 μg/ml) for 6 h. The supernatants were then incubated at 37°C for 10 min prior to performing the cytotoxicity assay with RPMI medium, rabbit anti-rmuTNF-α antibody or control antibody (serum from a non-immunized rabbit). Panel B: A sample of rmuTNF-α was used as a control for the TNF assay. Results are the mean ± S.E.M. of at least two experiments performed in quadruplicate. *$p < 0.01$ compared with control values (ANOVA; Duncan's test).

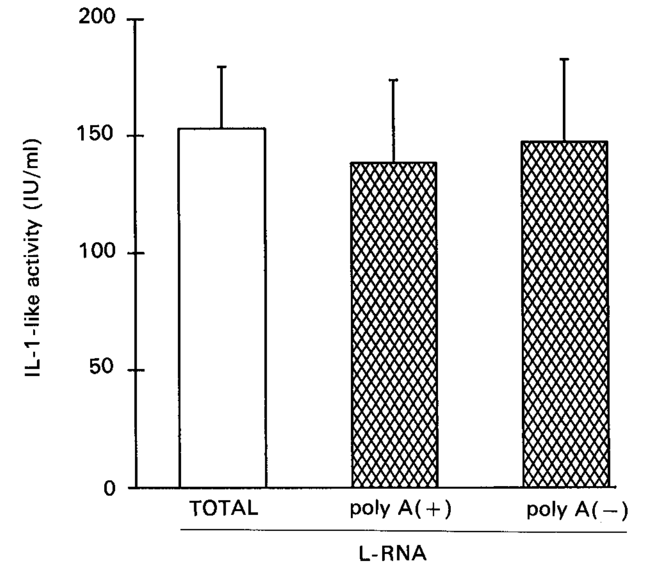

FIG. 3. The effect of the poly A(+)-RNA and poly A(−)-RNA fractions on the release of IL-1 by resident macrophages. Macrophage monolayers were incubated with total L-RNA (25 μg/ml), or with the poly A(+)-RNA (0.5 μg/ml) or poly A(−)-RNA fractions (25 μg/ml) for 6 h. Results are the mean ± S.E.M. of quadruplicates from a representative assay taken from three similar experiments.

stimulated macrophage monolayers was strongly reduced by an antiserum against TNF-α but not by the control serum. The effect of TNF-α antiserum to recombinant murine TNF-α is also shown in Fig. 2b.

The preincubation of macrophage monolayers with the inhibitors of protein synthesis cycloheximide (10 μg/ml) and actinomycin-D (10 μg/ml), or with dexamethasone (3 μg/ml), a general inhibitor of cytokine release, significantly blocked the secretion of TNF induced by L-RNA (Fig. 4a). The release of IL-1 by L-RNA stimulated macrophages was also inhibited by cycloheximide and by dexamethasone (Fig. 4b).

A radioactive material which precipitated with TCA (Fig. 5) and which was sensitive to RNAse (Fig. 6) was detected in the supernatants of macrophage monolayers preincubated with [5-$^3$H]-uridine and stimulated with LPS (10 μg/ml) or rIL-1β ($10^{-8}$ M). These properties suggest that the material was RNA. The release of radioactive RNA was not observed in macrophage monolayers stimulated with rTNF-α ($10^{-10}$–$10^{-8}$ M) or with LPS in doses of 0.1 or 1 μg/ml, or with IL-1β in doses $10^{-9}$ or $10^{-10}$ M (Fig. 5). rIL-6 and rIL-8 ($10^{-10}$–$10^{-8}$ M) were also ineffective in stimulating the release of RNA (data not shown). The sedimentation profile of the $^3$H-RNA released by LPS-stimulated or non-stimulated, macrophage monolayers in the sucrose gradient is shown in Fig. 7. The $^3$H-RNAs secreted under both conditions sediment in the same region of the gradient (4–5 S). However, there was an increase in $^3$H-RNA secretion by macrophages treated with LPS. Figure 7b shows that the $^3$H-RNA secreted by LPS stimulated macrophages was completely hydrolysed by KOH pretreatment. The sedimenta-

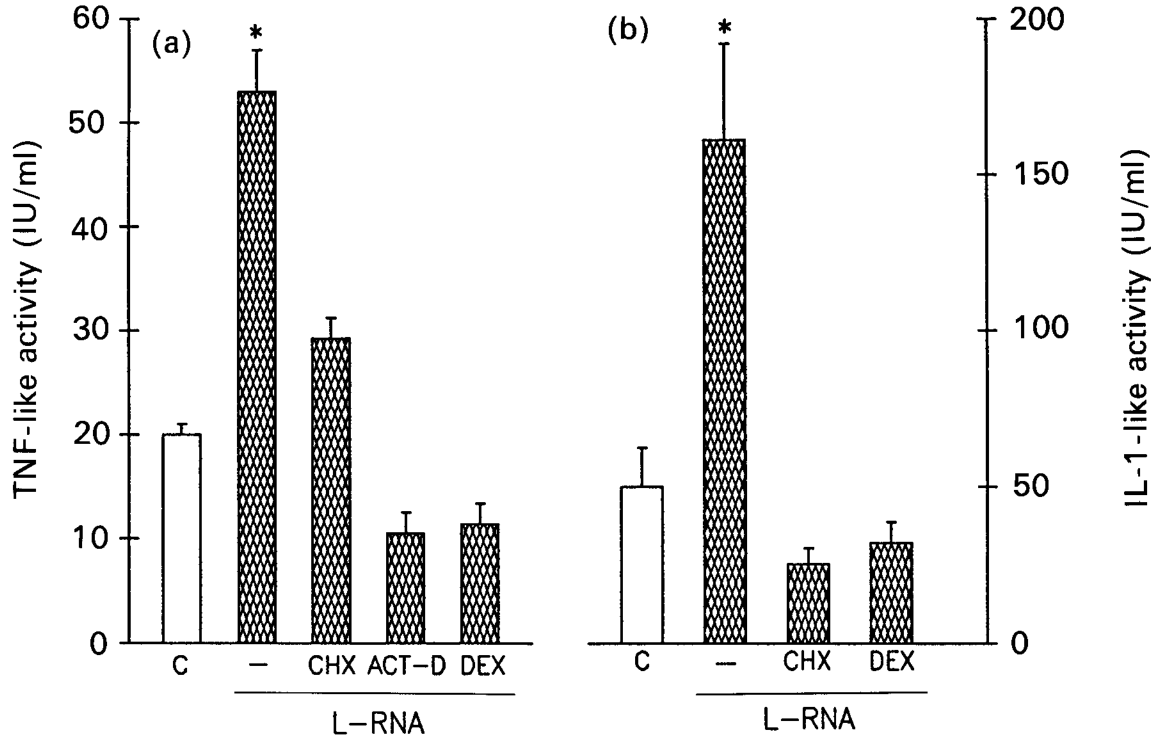

Fig. 4. The effect of pretreatment of resident macrophages with cycloheximide, actinomycin-D and dexamethasone on the release of TNF-α and IL-1 induced by L-RNA. Cycloheximide (CHX; 10 μg/ml), actinomycin-D (ACT-D; 10 μg/ml) or dexamethasone (DEX; 3 μg/ml) were added to RPMI medium 30 min before the addition of L-RNA (25 μg/ml). Incubation time was 6 h. The TNF-α activity (panel A) and the IL-1 activity (panel B) were quantified as described in Materials and Methods. Results are the mean ± S.E.M. of at least three experiments performed in quadruplicate. *$p < 0.01$ compared with control value (ANOVA; Duncan's test).

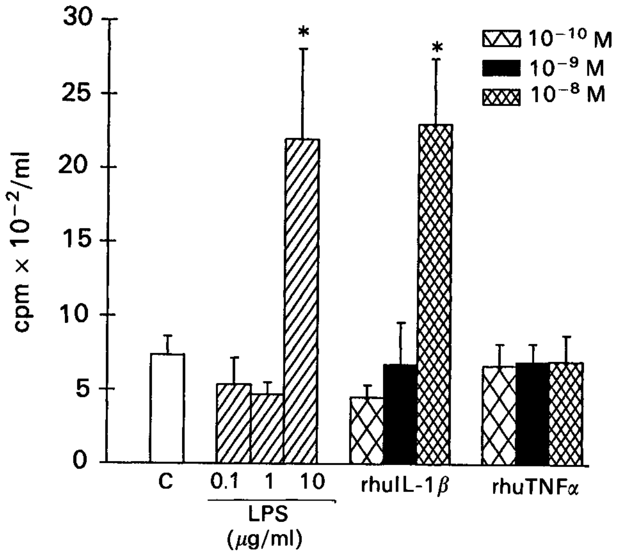

FIG. 5. The effect of LPS, rhuIL-1 and rhuTNF-α on the secretion of RNA by resident macrophages. Macrophage monolayers were preincubated with [5-$^3$H]-uridine (5 µCi/ml) for 2 h. This specific precursor of RNA synthesis was then removed from the cultures and the cells were incubated with LPS or recombinant human cytokines (rhuIL-1 and rhuTNF-α) for 6 h. The supernatants were treated with TCA and processed for $^3$H-RNA detection as described in Materials and Methods. The results (counts per minute; cpm) are expressed as the mean ± S.E.M. of experiments performed in quadruplicate. *$p < 0.05$ compared with control value (ANOVA; Duncan's test).

tion profile of the non-radioactive, cytoplasmic RNA is shown in Fig. 7a.

Incubation of the macrophage monolayers with LPS or IL-1β in concentrations that stimulated RNA secretion did not alter the viability of the macrophages. Table 1 shows that the LDH activity in the supernatants of macrophages before and after

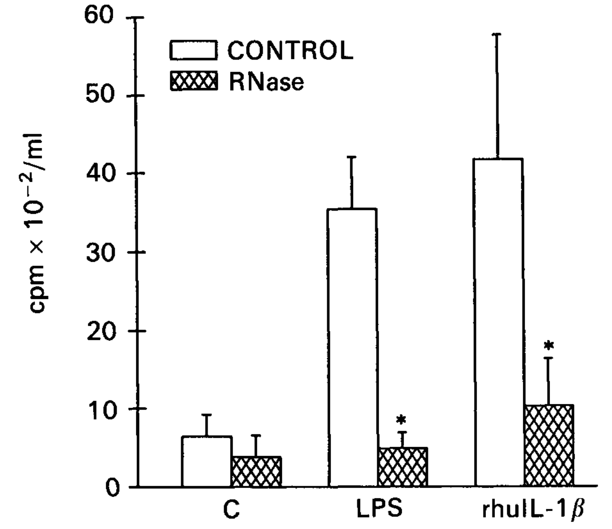

FIG. 6. The effect of RNase treatment of RNA secreted by macrophages stimulated with LPS and rhuIL-1. Macrophage monolayers were preincubated with [5-$^3$H]-uridine (5 µCi/ml) for 2 h. This specific precursor of RNA synthesis was then removed from the cultures and the cells were incubated with rhuIL-1 ($10^{-8}$ M) or LPS (10 µg/ml) for 6 h. The supernatants were incubated with RNase TCA and processed for $^3$H-RNA detection as described in Materials and Methods. The results (counts per minute, cpm) are expressed as the mean ± S.E.M. of three experiments performed in duplicate. *$p < 0.05$ compared with respective control. (ANOVA; Student's $t$ test).

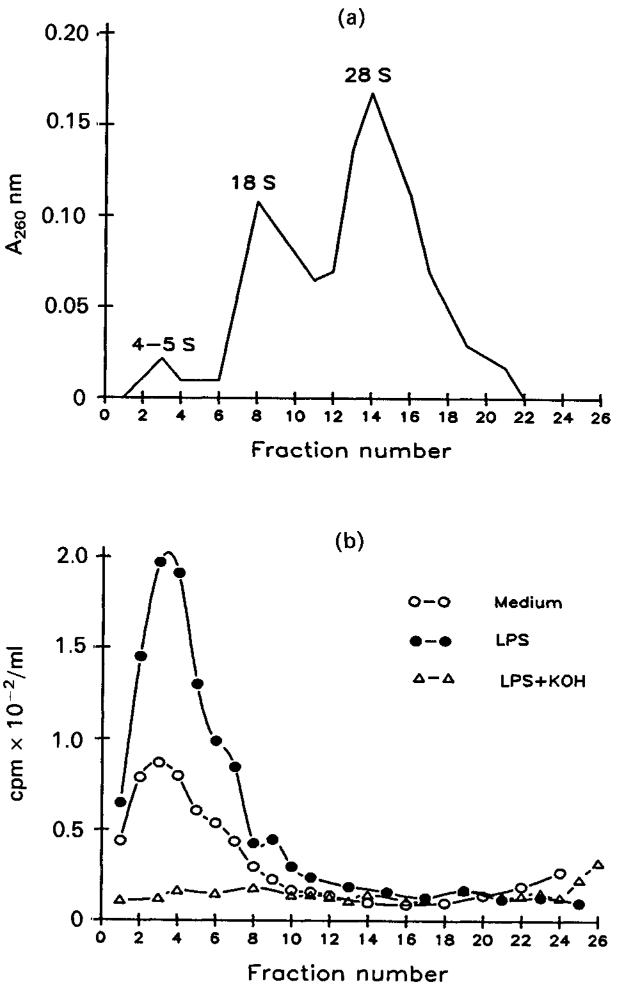

FIG. 7. Sedimentation profile of RNA secreted by normal or LPS-stimulated macrophages. Panel A: Sedimentation profile of non-radioactive RNA isolated from normal macrophages. Panel B: Sedimentation profile of RNA secreted by macrophage monolayers either unstimulated or stimulated with LPS (10 µg/ml). The RNA secreted by LPS stimulated macrophages was also hydrolyzed with KOH before centrifugation on a 5–20% sucrose gradient. The experimental conditions are given in Materials and Methods.

stimulation with LPS (10 µg/ml), IL-1β ($10^{-8}$ M) or TNF-α ($10^{-8}$ M) was not different from that observed in the supernatant of macrophages incubated with RPMI medium.

### Discussion

The present study shows that macrophage monolayers, when stimulated with L-RNA, release

**Table 1.** Detection of LDH activity in the supernatants of macrophage monolayers treated with LPS, rhuIL-1 and rhuTNF-α

| Treatment | LDH activity (units/ml/min) Before treatment | After treatment (6 h) |
|---|---|---|
| Medium | 0.173 | 0.184 |
| LPS (10 µg/ml) | 0.172 | 0.106 |
| rhuIL-1β ($10^{-8}$ M) | 0.161 | 0.172 |
| rhuTNF-α ($10^{-8}$ M) | 0.192 | 0.147 |

Data are the means of two experiments.

IL-1 and TNF-α into the supernatant, and that macrophages pre-incubated with [5-$^3$H]-uridine, a specific precursor of RNA synthesis, release $^3$H-RNA into the culture supernatant when stimulated with LPS or IL-1β. These data suggest that exogenous RNA may mediate secretion of cytokines by resting macrophages.

The L-RNA-induced release of cytokines by macrophages is assumed to be RNA specific and not due to LPS contamination, because: (a) this effect is strongly reduced by ribonuclease; (b) N-RNA has no biological effects, thus indicating that the polynucleotide chain by itself is not able to induce cytokine release; and (c) polymyxin B, in a dose that inhibits LPS-induced release of IL-1 and TNFα, does not modify L-RNA activity.

There is much evidence showing that poly A(+)-RNA is the main active biological fraction of exogenous RNA[11,20,26,27] despite some findings showing a functional effect for poly A(−)-RNA.[28] In an attempt to identify the fraction of L-RNA active in cytokine release, total cytoplasmic L-RNA samples were fractionated by oligo (dT)–cellulose chromatography and the activity of the poly A(+)- and poly A(−)-RNA fractions on IL-1 and TNF-α release were studied. The poly A(+)-RNA (0.5 μg/ml), poly A(−)-RNA (25 μg/ml) and total L-RNA (25 μg/ml) had similar effects on the release of the two cytokines.

It is known that poly A(+) containing RNA is the messenger RNA fraction and comprises about 1–2% of the total cellular RNA of eukaryotic cells.[19] In our experiments the dose of poly A(+) that induced the release of TNF-α and IL-1 (0.5 μg/ml) corresponds to 2% of the dose of the total L-RNA that also induced cytokine release. This finding is strong evidence that at least part of the L-RNA acts as informational RNA. Corroborating this idea is the fact that cycloheximide, an inhibitor of RNA translation in eukaryotic cells,[29] inhibited the release of TNF-α and IL-1 by L-RNA-stimulated macrophages. The release of these cytokines was also found in the poly A(−)-RNA treated macrophages, suggesting that L-RNA may also act at the transcriptional level. The inhibition of L-RNA activity by actinomycin-D, a classical inhibitor of transcription, confirms this assumption.

It is known that the release of cytokines stimulated by different stimuli is strongly inhibited by glucocorticoids acting at the transcriptional and/or translational levels.[30–32] In our experiments dexamethasone blocked the release of IL-1 and TNF from the L-RNA stimulated macrophages which is additional evidence that L-RNA may act at the transcriptional and/or translational levels.

It is now accepted that resident macrophages may act as alarm cells.[33] These cells are highly reactive and, when activated by various stimuli, secrete several mediators including cytokines, which play an important role in triggering and maintaining the defence response.[14,15] In the first part of this paper the authors show that the RNA obtained from LPS-activated macrophages can mediate cytokine release. In the second part of this work, it is shown that these macrophages, when pre-incubated with [5-$^3$H]-uridine and activated with LPS, released a radioactive, TCA-precipitable material into the supernatant which was hydrolysed by RNase and KOH. It is assumed therefore, that these radioactive macromolecules represent RNA which sediments in the 4–5 S region of the sucrose gradient. There are a few reports in the literature[24,34,35] concerning the release by eukaryotic cells of RNA which sediments in the 2.5–5.0 S region of the sucrose gradient. The present observation is the first demonstration that eukaryotic cells release RNA after an exogenous stimulus such as LPS, since previous reports concern spontaneously released RNA.

One interesting finding was that RNA secretion is also induced by an endogenous, pro-inflammatory mediator, IL-1β, but not by IL-6, IL-8 or TNF-α, suggesting that the release is mediator specific. That RNA secretion may be due to cellular damage was eliminated by the demonstration of cell viability by eosin Y exclusion and by the measurement of lactate dehydrogenase activity in the supernatants of the LPS- and cytokine-stimulated macrophages. Together, the facts that RNA obtained from activated macrophages can stimulate cytokine release by resting macrophages, and that activated macrophages also release RNA, provide experimental evidence for the concept that RNA may be an intercellular mediator. To obtain further insight concerning the implications of RNA secretion, the biological activity of such secreted RNA is currently under investigation.

In conclusion, our data suggest that resident macrophages, when stimulated by injurious stimuli, in addition to secreting cytokines, are also able to release RNA which may act on the surrounding, resting macrophages, thus stimulating the release of more cytokines. This process may represent amplification of the mechanisms involved in defence responses.

### References

1. Gallagher RE, Walter CA, Gallo RC. Uptake and aminoacylation of exogenous transfer RNA by mouse leukemia cell. *Biochem Biophys Res Commun* 1972; **49**: 782–792.
2. Wang SR, Giacomoni D, Dray S. Physical and chemical characterization of RNA incorporated by rabbit spleen cells. *Exp Cell Res* 1973; **78**: 15–24.
3. McLean MJ, Renaud JF, Niu MC, Sperelakis N. Membrane differentiation of cardiac myoblasts induced *in vivo* by an RNA-enriched fraction from adult heart. *Exp Cell Res* 1977; **110**: 1–14.
4. Slomski R, Latos AM. Uptake of immune RNA by normal mouse spleen cells. *Mol Cell Biochem* 1979; **24**: 15–20.
5. Mroczkowski B, Dym HP, Siegel EJ, Heywood SM. Uptake and utilization of RNA by myogenic cells in culture. *J Cell Biol* 1980; **87**: 65–71.
6. Davis LA, Lemanski LF. Induction of myofibrillogenesis in cardiac lethal mutant axolotl hearts rescued by RNA derived from normal endoderm. *Development* 1987; **99**: 145–154.

7. Zucchi TMAD, Passos Jr GAS, De Lucca FL. RNA-mediated genetic transformations in *Aspergillus nidulans*. *Cell Mol Biol* 1989; **35**: 573–580.
8. Passos Jr GAS, De Lucca FL. RNA-mediated transfer of cellular immunity to a synthetic env antigen of the human immunodeficiency virus (HIV-1). *Mol Cell Biochem* 1991; **108**: 1–8.
9. Wolff JA, Malone RW, Williams P *et al.* Direct gene transfer into mouse muscle *in vivo*. *Science* 1990; **247**: 1465–1468.
10. Sokol S, Christian JL, Moon RT, Melton DA. Injected Wnt RNA induces a complete body axis in *Xenopus* embryo. *Cell* 1991; **67**: 741–752.
11. Passos Jr GAS, De Lucca FL. *In vivo* induction of immunological memory to human tumor extract with poly (A)-containing immune RNA. *Cell Mol Biol* 1988; **34**: 157–164.
12. Unanue ER, Allen PM. The basis for immunoregulatory role of macrophages and other accessory cells. *Science* 1987; **236**: 551–557.
13. Nathan CF. Secretory products of macrophages. *J Clin Invest* 1987; **79**: 319–326.
14. Larrick JW, Kunkel SL. The role of tumor necrosis factor and interleukin 1 in the immunoinflammatory response. *Pharmaceut Res* 1988; **5**: 129–139.
15. Mizel SB. The interleukins. *FASEB J* 1989; **3**: 2379–2388.
16. Akira S, Hirano T, Taga T, Kishimoto T. Biology of multifunctional cytokines: IL-6 and related molecules (IL-1 and TNF). *FASEB J* 1990; **4**: 2860–2867.
17. Arai K, Lee F, Miyajima A, Miyatake S, Arai N, Yokota T. Cytokines: coordinators of immune and inflammatory responses. *Ann Rev Biochem* 1990; **59**: 783–836.
18. Benner SA. Extracellular 'Communicator RNA'. *FEBS Lett* 1988; **233**: 225–228.
19. White BN, De Lucca FL. Preparation and analysis of RNA. In: Tunner RB, ed. *Analytical Biochemistry of Insects*. Amsterdam: Elsevier Scientific, 1977; 85–130.
20. Bertolini MC, De Lucca FL. Poly (A)-containing RNA from the spleens of mice with Chagas' disease triggers *in vitro* macrophage resistance to *Trypanosoma cruzi*. *J Protozool* 1986; **33**: 81–84.
21. Aviv H, Leder P. Purification of biologically active globin messenger RNA by chromatography on oligothymidilic acid–cellulose. *Proc Natl Acad Sci USA* 1972; **69**: 1408–1412.
22. Espevik T, Nissen-Meyer J. A highly sensitive cell line, WEHI 164 clone 13 for measuring cytotoxic factor/tumor necrosis factor from human monocytes. *J Immunol Methods* 1986; **95**: 99–105.
23. Gery I, Gerhson RK, Waksman BH. Potentiation of the T-lymphocyte response to mitogens. I-Responding cell. *J Exp Med* 1972; **136**: 128–134.
24. Kolodny GM, Culp LA, Rosenthal LJ. Secretion of RNA by normal and transformed cells. *Exp Cell Res* 1972; **73**: 65–72.
25. Bergmeyer HU, Bernt E. Lactate dehydrogenase. In: Bergmeyer HU, ed. *Methods of Enzymatic Analysis, volume 2*. New York: Academic Press, Inc., 1974; 574–579.
26. Wang BS, Mannick JA. Fractionation of immune RNA capable of transferring tumor-specific cellular cytotoxicity. *Cell Immunol* 1978; **37**: 358–368.
27. Greenup CJ, Vallera DA, Pennline KJ, Kolodziej BJ, Dodd MC. Antitumor cytotoxicity of poly (A)-containing messenger RNA isolated from tumor-specific immunogenic RNA. *Br J Cancer* 1978; **38**: 55–63.
28. Kodama K, Yoshida T, Shibuya T, Kurashige S, Mitsuhashi S. Induction of IgM memory with RNA from the spleens of immunized mice. *Immunology* 1981; **44**: 535–542.
29. Stewart PR. Inhibitors of translation. In: Stewart PR, Letham DS, eds. *The Ribonucleic Acids*. Berlin: Springer-Verlag, 1973; 151–158.
30. Beutler B, Krochin N, Milsark IW, Luedke C, Cerami A. Control of cachetin (tumor necrosis factor) synthesis: mechanisms of endotoxin resistance. *Science* 1986; **232**: 977–980.
31. Lew W, Oppenheim JJ, Matsushima K. Analysis of the suppression of IL-1 alpha and IL-1 beta production in human peripheral blood mononuclear adherent cells by a glucocorticoid hormone. *J Immunol* 1988; **140**: 1895–1902.
32. Waage A, Bakke O. Glucocorticoids suppress the production of tumour necrosis factor by lipopolysaccharide-stimulated human monocytes. *Immunology* 1988; **63**: 299–302.
33. Ferreira SH. Are macrophages the body's alarm cells? *Agents Actions* 1980; **10**: 229–230.
34. Kolodny GM. Evidence for transfer of macromolecular RNA between mammalian cells in culture. *Exp Cell Res* 1971; **65**: 313–324.
35. Stroun M, Anker P, Beljanski M, Henri J, Lederrey C, Ojha M, Maurice PA. Presence of RNA in the nucleoprotein complex spontaneously released by human lymphocytes and frog auricles in culture. *Cancer Res* 1978; **38**: 3546–3554.

ACKNOWLEDGEMENTS. R. A. Ribeiro was on leave from the Department of Physiology and Pharmacology, Faculty of Medicine, University of Ceará, Ceará, Brazil. C. A. Flores was on leave from the Department of Pharmacology, Faculty of Biological Sciences, University of Campinas – UNICAMP, Campinas, Brazil. R. A. Ribeiro and C. A. Flores were supported by a fellowship from Conselho Nacional de Pesquisa Científica-CNPq. The authors gratefully acknowledge the technical assistance of Sérgio Roberto Rosa and Cacilda Dias Perreira Zanon. This work was supported by FAPESP and CNPq (Brazil).

**Received 16 August 1993; accepted in revised form 28 September 1993**
